# KDM4A regulates HIF-1 levels through H3K9me3

**DOI:** 10.1038/s41598-017-11658-3

**Published:** 2017-09-11

**Authors:** Grzegorz Dobrynin, Tom E. McAllister, Katarzyna B. Leszczynska, Shaliny Ramachandran, Adam J. Krieg, Akane Kawamura, Ester M. Hammond

**Affiliations:** 1 Cancer Research UK and Medical Research Council Oxford Institute for Radiation Oncology, Department of Oncology, The University of Oxford, Oxford, OX3 7DQ UK; 2 Division of Cardiovascular Medicine, Radcliffe Department of Medicine, Wellcome Trust Centre of Human Genetics, Roosevelt Drive, The University of Oxford, Oxford, OX3 7BN UK; 3 Department of Chemistry, Chemistry Research Laboratory, The University of Oxford, Mansfield Road, Oxford, OX1 3TA UK; 4 Department of Obstetrics and Gynecology, Oregon Health & Science University, Portland, Oregon, USA

## Abstract

Regions of hypoxia (low oxygen) occur in most solid tumours and cells in these areas are the most aggressive and therapy resistant. In response to decreased oxygen, extensive changes in gene expression mediated by Hypoxia-Inducible Factors (HIFs) contribute significantly to the aggressive hypoxic tumour phenotype. In addition to HIFs, multiple histone demethylases are altered in their expression and activity, providing a secondary mechanism to extend the hypoxic signalling response. In this study, we demonstrate that the levels of HIF-1α are directly controlled by the repressive chromatin mark, H3K9me3. In conditions where the histone demethylase KDM4A is depleted or inactive, H3K9me3 accumulates at the HIF-1α locus, leading to a decrease in HIF-1α mRNA and a reduction in HIF-1α stabilisation. Loss of KDM4A in hypoxic conditions leads to a decreased HIF-1α mediated transcriptional response and correlates with a reduction in the characteristics associated with tumour aggressiveness, including invasion, migration, and oxygen consumption. The contribution of KDM4A to the regulation of HIF-1α is most robust in conditions of mild hypoxia. This suggests that KDM4A can enhance the function of HIF-1α by increasing the total available protein to counteract any residual activity of prolyl hydroxylases.

## Introduction

Hypoxia occurs in most solid tumours as a consequence of the rapid proliferation of cancer cells and an inadequate/inefficient vasculature. Most importantly, the degree of tumour hypoxia has been shown to correlate with poor patient survival in numerous tumour types^1^. The predominant transcriptional response to hypoxia is mediated by the hypoxia inducible factors (HIF), which include HIF-1, 2 and 3^2, 3^. Each HIF is composed of the same constitutively expressed β subunit (HIF-1β) and an oxygen labile α subunit (HIF-1α, 2α or 3α). Under normoxic conditions, HIF-1α is hydroxylated by the prolyl hydroxylases (PHDs) within the N- and C- terminal oxygen dependent degradation domains (NODD and CODD)^4^. Once hydroxylated, HIF-1α interacts with the tumour suppressor, von-Hippel-Lindau complex (VHL), is ubiquitinated and targeted for proteosomal degradation. The PHDs are Fe(II) and 2-oxoglutarate (2-OG) dependent oxygenases and do not function in hypoxic conditions where insufficient oxygen is available, therefore allowing the stabilisation of HIF-α subunits and dimerisation with HIF-1β^5^. HIF-1α function is also regulated by another 2-OG oxygenase, factor inhibiting HIF (FIH). FIH hydroxylates an asparagine residue in the C-terminal transactivation domain of HIF1-α which prevents complex formation with p300, thus inhibiting transcriptional activation^6^. In addition to HIF-mediated changes in gene expression, the epigenetic landscape is also altered in response to hypoxia^7, 8^. KDM4A (JMJD2A) is a member of the histone lysine demethylase (KDM) family of enzymes that catalyse the removal of methyl groups from lysine residues and are involved in transcriptional regulation of gene expression^9–11^. Like the PHDs, the enzymatic activity of KDMs relies on 2-OG, Fe(II) and the presence of molecular oxygen as essential cofactors^12–15^. KDM4A expression has been reported to be increased in several cancer types including colorectal, this combined with a number of functional studies have suggested KDM4A is an attractive target for cancer therapy^16–20^. In this study, we describe a novel role for KDM4A in the regulation of HIF-1α mRNA expression and identify inhibition of KDM4A as a unique strategy to decrease HIF signalling. These findings strongly support the hypothesis that KDM4A is a potential therapeutic target for improving the treatment response of radioresistant, hypoxic solid tumours.

## Results

### KDM4A as a therapeutic target

**Figure 1:**
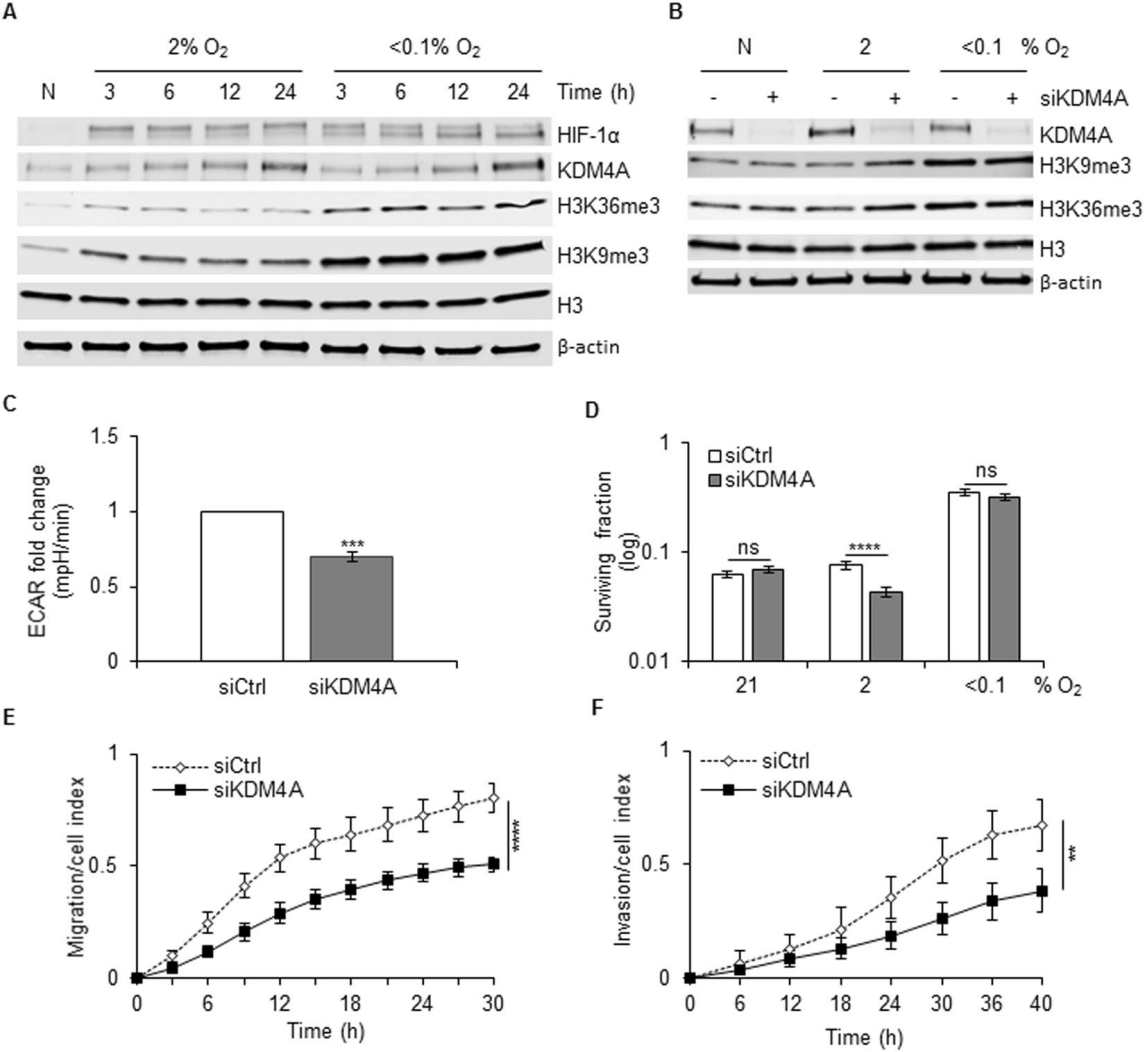
Biological consequences of siRNA-mediated KDM4A depletion. (**A**) RKO cells were exposed to the O_2_ concentrations indicated for the periods of time shown. N (normoxia) is 21% O_2_ for 24 h. Western blotting was carried out with the indicated antibodies. (**B**) RKO cells were transfected with siRNA to KDM4A (siKDM4A) or a non-targeting control siRNA and then incubated at 21%, 2% or <0.1% O_2_ for 24 h. Uncropped blots for the Fig. 1A and B are presented in Figure S5A and B. (**C**) ECAR was measured in RKO cells treated with siKDM4A or siCtrl. (**D**) RKO cells were treated with siKDM4A and then incubated in 2% O_2_ for 24 h and irradiated with 6 Gy while in hypoxic conditions (Supplementary Figure S1E)^41^. A colony survival assay is shown. (**B**) MDA-MB-231 cells were treated with siKDM4A or siCtrl and migration measured in 2% O_2_ over a period of 30 h. (**F**) MDA-MB-231 cells were treated with siKDM4A or siCtrl and invasion measured in 2% O_2_ over a period of 40 h.

KDM4A has been described as over-expressed in a range of cancer types although the underlying mechanism is unclear. As both KDM4B and KDM4C have been demonstrated to be targets of HIF-1, we investigated the possibility that KDM4A is also hypoxia regulated by correlating expression with a previously described hypoxia signature^21–23^. Using the TCGA colorectal adenocarcinoma data set, we found no significant correlation between KDM4A expression and the hypoxia signature. In contrast, KDM4B expression did positively correlate with the hypoxia signature (Supplementary Figure S1A,B). In support of this finding there was no increase in KDM4A mRNA expression after exposure to hypoxia (2% or <0.1% O_2_) (Supplementary Figure S1C). However, in agreement with previous reports suggesting KDM4A has a pro-longed half-life in hypoxia, there was a clear increase in KDM4A protein levels in RKO cells exposed to either 2% or <0.1% O_2_, (Fig. 1A)^24, 25^. As expected, the levels of H3K9me3 and H3K36me3 increased in response to <0.1% O_2_ (Fig. 1B)^26, 27^. Interestingly, although the effect of depletion of KDM4A on the total levels of H3K9me3 and H3K36me3 was slight, this was most pronounced at 2% O_2_ (Fig. 1B and Supplementary Figure S1D). Since KDM4A has been described as having the potential to act as an oxygen sensor and is highly sensitive to oxygen concentration, it is likely that while KDM4A would retain activity in our mild hypoxic conditions (2% O_2_), it would be inactive at the more severe level (<0.1% O_2_)^28^. To evaluate KDM4A as a potential therapeutic target we examined the KDM4A-dependent contribution to key biological processes associated with cancer aggressiveness. Depletion of KDM4A significantly decreased the extracellular acidification rate (ECAR) in RKO cells exposed to hypoxia, which is representative of the glycolytic rate (Fig. 1C). Next, we asked if loss of KDM4A affected the radiosensitivity of cells in hypoxic conditions (2 and <0.1% O_2_). The radiosensitivity of RKO cells was unaffected by loss of KDM4A when cells were in normoxic or severely hypoxic (<0.1% O_2_) conditions, although as expected the cells in <0.1% O_2_ were significantly more radioresistant (Supplementary Figure S1E,F and G). However, there was a modest but significant increase in radiosensitivity when KDM4A was depleted from cells exposed to 2% O_2_ during radiation (Fig. 1D and Supplementary Figure S1G). We also examined the migration rate of the highly motile cell line, MDA-MB-231, depleted of KDM4A. As previously reported the migration rate of KDM4A-depleted cells in 21% O_2_ was lower, although in our hands this was not a significant decrease (Supplementary Figure S1I)^29^. However, in hypoxic conditions (2% O_2_) depletion of KDM4A led to a significant decrease in migration rate (Fig. 1E). Finally, depletion of KDM4A also led to a decreased invasion rate under hypoxic conditions (2% O_2_) (Fig. 1F). Together, these data support the hypothesis that KDM4A is a good candidate for therapeutic intervention and that this might be particularly effective as a means of targeting the radioresistant, hypoxic fraction of solid tumours.

### Off-target effects of pharmacological KDM4A inhibitors can stabilise HIF-1α

**Figure 2:**
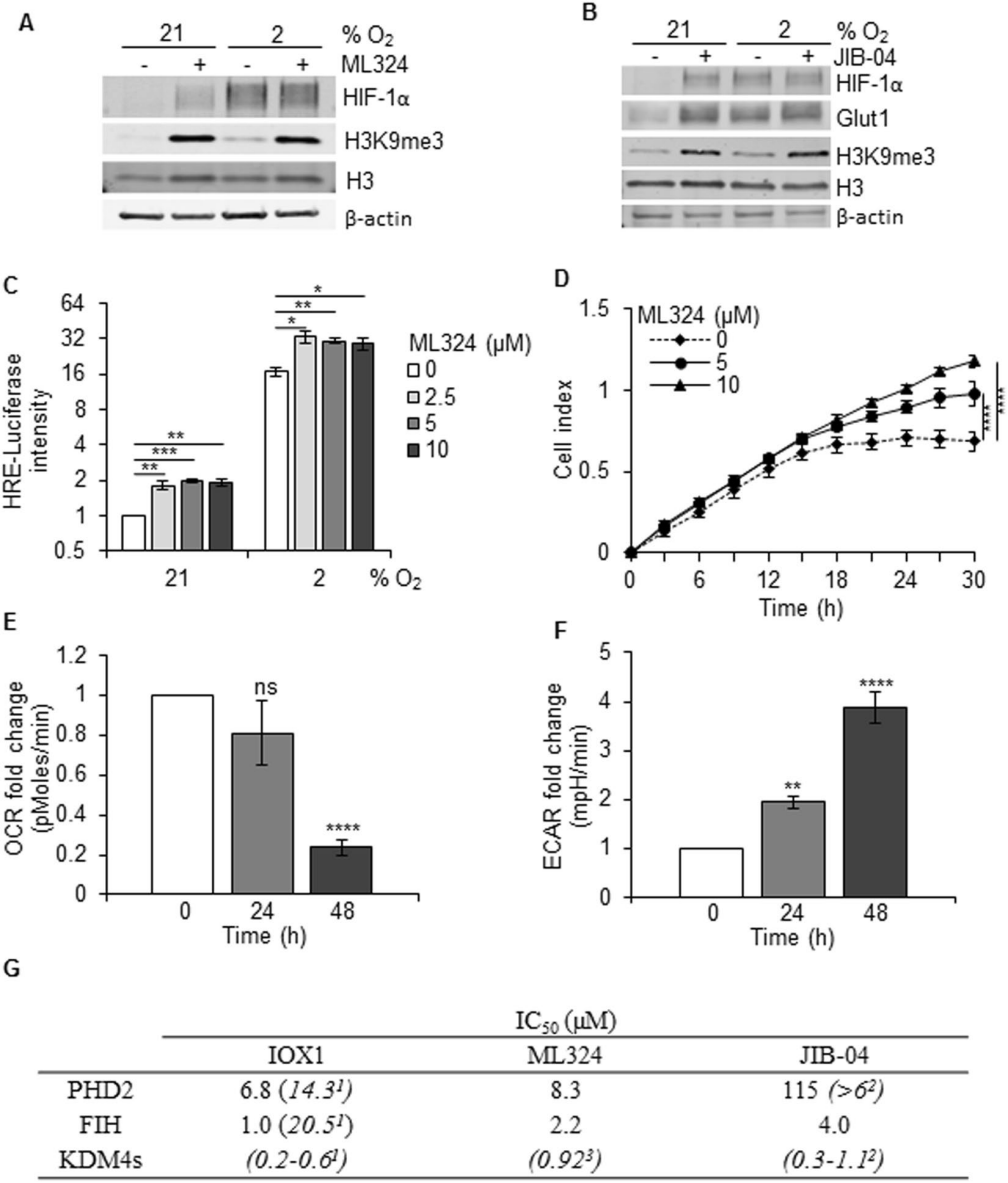
ML324 stabilises HIF-1α protein levels in normoxic conditions (21% O_2_). (**A**) RKO cells were treated with ML324 (10 µM) for 48 h in either 21% or 2% O_2_. Western blotting was then carried out. (**B**) RKO cells were treated as in (**A**) with JIB-04 (5 µM). Uncropped blots are shown in Figure S6A and B. (**C**) RKO cells co-transfected with HRE (HIF-1 responsive element)-Firefly Luciferase and Renilla Luciferase were treated with ML324 (10 µM) for 24 h, incubated in 21% O_2_ or 2% O_2_ for an additional 24 h and the intensity of Firefly Luciferase was measured relative to the levels of Renilla Luciferase. (**D**) The relative motility of MCF-7 cells treated with ML324 was determined by xCELLigence assay. (**E**) Oxygen consumption rate (OCR) of RKO cells treated with ML324 (10 µM) for the indicated periods of time was measured. (**F**) Extracellular acidification rate (ECAR) of RKO cells treated with ML324 (10 µM) for the indicated periods of time was measured. (**G**) IC_50_ determination of inhibitors against PHD2 and FIH using mass spectrometry assay. Italicised IC_50_ values are reported values from literature^30–32^. The recombinant proteins used were PHD2_181-426_^43^ and recombinant FIH as previously described^44^. Dose response curves were generated as shown in Supplementary Figure S2G and H and used to calculate the IC_50_ values shown.

To pursue a more clinically relevant method of KDM4A inhibition we employed the recently described inhibitor ML324, which is a small molecule displaying submicromolar inhibitory activity toward the JMJD2/KDM4 family^30^. As expected, when RKO cells were exposed to ML324 in either normoxic or hypoxic (2% O_2_) conditions the levels of H3K9me3 increased significantly. Surprisingly, cells treated with ML324 in normoxic conditions had increased levels of HIF-1α (Fig. 2A and Supplementary Figure S2A,B). This finding was also observed in three oesophageal cancer cell lines (Supplementary Figure S2C). To determine whether this was due to a specific ML324 off-target effect, we used a second inhibitor, JIB-04, which inhibits the activity of the KDM family of histone demethylases, including KDM4A^31^. Again, HIF-1α levels were increased in response to the KDM4A inhibitor, and there was evidence that this led to a significant HIF-mediated response as the HIF-target Glut1 was also increased (Fig. 2B). We also used IOX1, a broad-spectrum, cell permeable inhibitor of most 2-OG oxygenases, including many members of the KDM demethylase family as well as PHD and FIH hydroxylases^32^. As expected, treatment of the RKO cells with IOX1 led to stabilisation of HIF-1α in normoxic conditions (Supplementary Figure S2D). In accordance with the normoxic stabilisation of HIF-1α in ML324-treated cells, we also observed a modest but significant increase in the HIF-1α target genes, Glut1 and VEGF in normoxia (Supplementary Figure S2E). This ML324-dependent increase in VEGF expression was also evident in hypoxic conditions (2% O_2_) (Supplementary Figure S2F). These data suggest that the HIF-1α stabilised in response to ML324 was active and sufficient to impact gene expression in both normoxia and hypoxia. This was further supported through the use of a luciferase reporter assay (Fig. 2C). Furthermore, treatment with ML324 increased cell motility in normoxia (Fig. 2D), decreased the oxygen consumption rate (OCR) (Fig. 2E) and increased ECAR (Fig. 2F). Together these data demonstrate that ML324 does not mimic the biological consequences of depletion of KDM4A. *In vitro* characterisation of the inhibitory activity of ML324 against recombinant HIF hydroxylase enzymes PHD2 and FIH revealed that ML324 inhibits the HIF hydroxylases at micromolar range (Fig. 2G, Supplementary Figure S2G and H). Therefore, it is likely that ML324, like the broad-spectrum 2OG oxygenase inhibitor IOX1, also inhibits HIF hydroxylases in cells resulting in up regulation of HIF. Also, we cannot rule out other off-target effects in the cellular context. Interestingly, JIB-04 is a very weak inhibitor of PHD2 (IC_50_ > 100 μM) but shows HIF up regulation in cells. While inhibition of other PHD isoforms cannot be ruled out, HIF stabilisation by these inhibitors may also arise from indirect effect of cellular PHD inhibition, via changes in iron availability as previously observed for metal-chelating 2OG oxygenase inhibitor scaffolds^33^. More KDM4 selective inhibitors are therefore needed in order to pharmacologically validate KDM4A as a therapeutic target^34^.

### Depletion of KDM4A decreases the HIF-1 response in hypoxia

**Figure 3:**
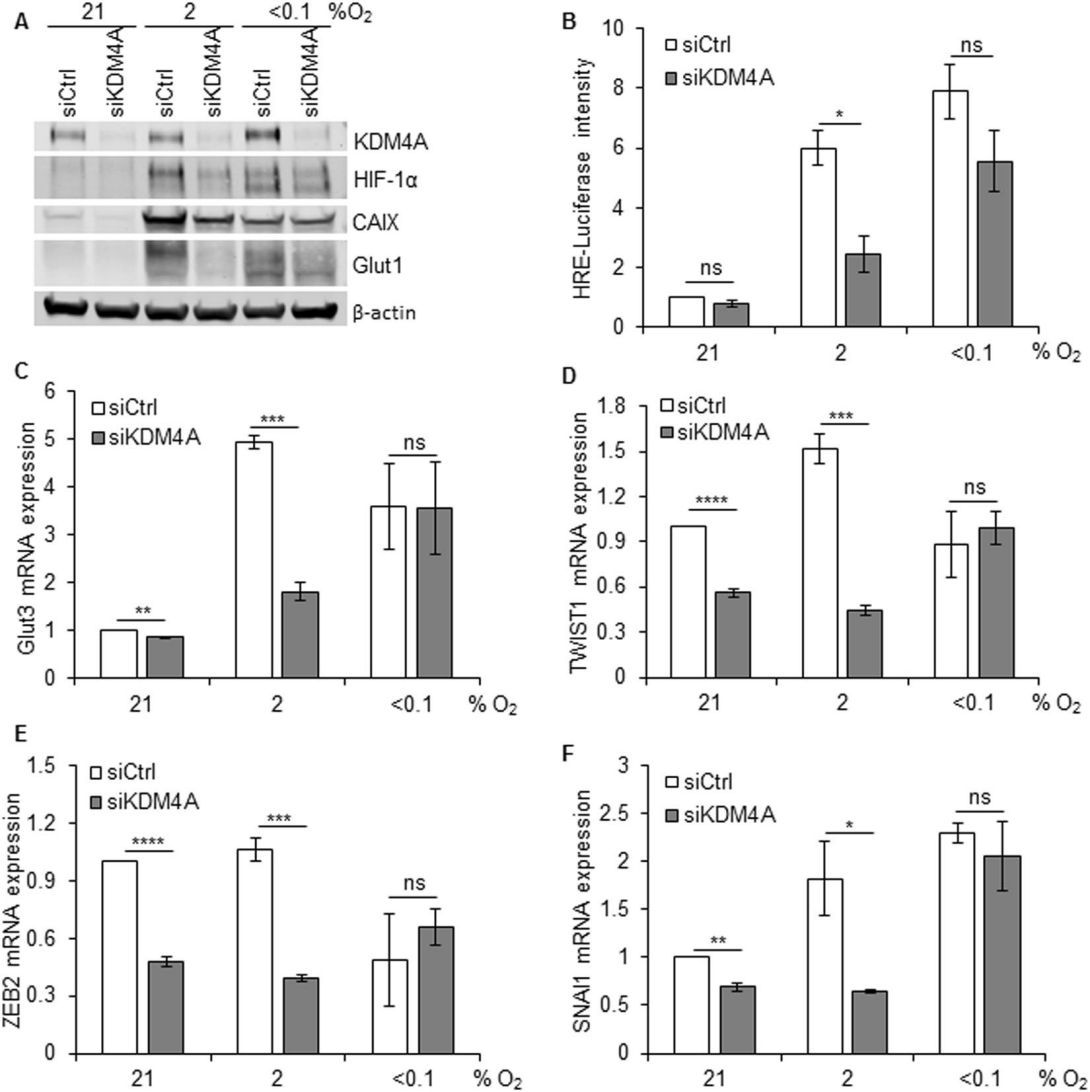
Depletion of KDM4A leads to decrease of HIF-1α activity in hypoxia (2% O_2_). (**A**) RKO cells were treated with siKDM4A and then incubated in 21%, 2% or < 0.1% O_2_ for 24 h. Western blotting was carried out. Uncropped blots are shown in Figure S7. (**B**) FaDu^HRE-Luc^ cells were treated as in part (**A**) and the relative intensity of Firefly Luciferase was measured relative to the number of cells in the respective conditions. RKO cells were treated as in part (**A**) and then mRNA levels were determined; (**C**) Glut3, (**D**) TWIST1, (**E**) ZEB2 and (**F**) SNAI1.

In contrast to ML324/JIB-04 treatment, depletion of KDM4A using siRNA led to a significant decrease in HIF-1α levels in hypoxia. This decrease in HIF-1α was more significant in cells exposed to 2% O_2_ compared to <0.1% O_2_ following KDM4A depletion (Fig. 3A). The levels of CAIX and Glut1, both HIF-1 targets, were also decreased in a KDM4A-dependent manner. Decreased HIF-1α after siRNA-mediated depletion of KDM4A was verified using 4 additional siRNAs to validate that this was not due to an off-target effect and was also verified in additional cell lines (MDA-MB-231 and HCT116) (Supplementary Figure S3A,B and C). Interestingly, a decrease in HIF-2α was also observed in HCT116 cells exposed to hypoxia (2% O_2_) upon depletion of KDM4A (Supplementary Figure S3C). Decreased HIF-1 activity in hypoxia after KDM4A depletion was further confirmed using a reporter assay (Fig. 3B) and by measuring the expression of specific HIF target genes; Glut 1 (Supplementary Figure S3D), CAIX (Supplementary Figure S3E), SLC2A3/Glut3, which regulates metabolism (Fig. 3C) and TWIST1, which has a role in cell migration (Fig. 3D). In each case loss of KDM4A leads to decreased induction of the HIF-target gene in response to hypoxia (2% O_2_). Loss of KDM4A did not impact gene expression in <0.1% O_2_ and we attribute this to the oxygen dependency of KDM4A and suggest that as the demethylase would be unable to function at <0.1% O_2_, there was no added effect of reducing the level of KDM4A protein. To ensure that loss of KDM4A had not reduced transcription in general we measured the expression of a non-hypoxia responsive gene, OAZ1, and found this to be unaffected by KDM4A loss (Supplementary Figure S3F). Interestingly, recent reports describe a novel mechanism in which KDM4A interacts with and co-activates the transcription factors E2F1 and ETV1, which have roles in regulating cell metabolism and migration^20, 35^. ZEB2 is a known target of E2F1 and HIF-1 and so we investigated the KDM4A-dependent effects on expression in both normoxia and hypoxia^35^. ZEB2 was not induced in response to hypoxia in the RKO cells used, however loss of KDM4A significantly decreased ZEB2 expression in both normoxia and hypoxia (2% O_2_) (Fig. 3E). Loss of KDM4A had a similar effect on SNAI1 expression, which is regulated by ETV1 and HIF-1 (Fig. 3F). It is likely that the KDM4A-dependent effects on ZEB2 and SNAI1 expression observed in normoxia could be attributed to decreased activity of E2F1 and ETV1 respectively, whilst the effects seen in hypoxia may result from a combination of repressed E2F1/ETV1 and HIF-1 activity. Again, there was no significant impact of depleting KDM4A on gene expression in the more severe hypoxia (<0.1% O_2_).

### HIF-1α mRNA expression is regulated by H3K9me3

**Figure 4:**
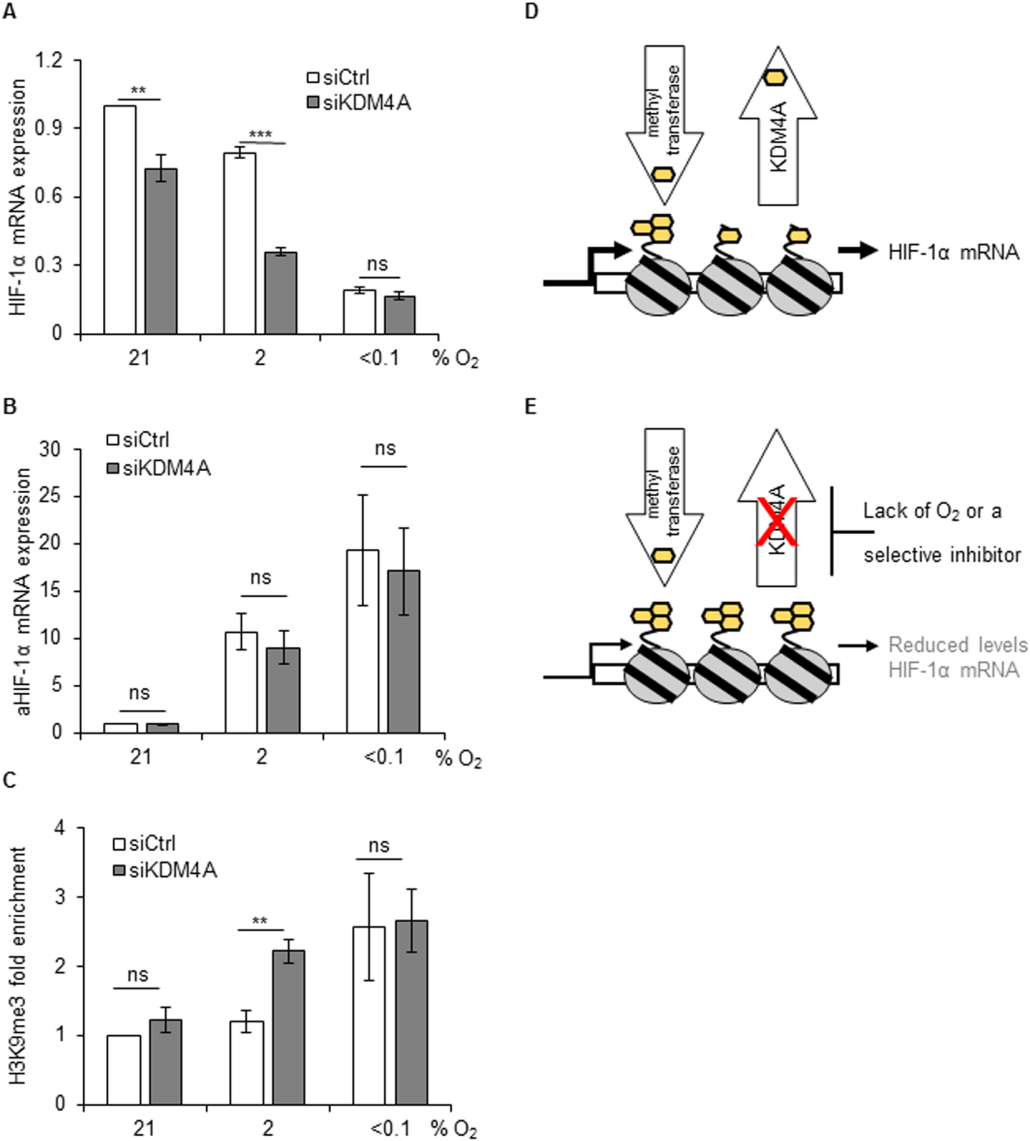
Mechanism of KDM4A-dependent effect on HIF-1. RKO cells were treated with siKDM4A and then incubated in 21%, 2% or < 0.1% O_2_ for 24 h. qRT-PCR for HIF-1α (**A**) or aHIF-1α (**B**) is shown. (**C**) Cells were treated as in (**A**), then ChIP for H3K9me3 was performed and the enrichment of H3K9me3 on the *HIF-1A* gene relative to H3 in response to hypoxia were measured^26^. A schematic to demonstrate the regulation of HIF-1α by KDM4A. In normoxic or mildly hypoxic (2% O_2_) conditions the level of H3K9me3 associated with the *HIF-1Α* gene are low due to the activity of the methyl transferase and KDM4A (**D**). However, if KDM4A is depleted or inactivated due to lack of an essential co-factor (O_2_) or potentially a specific inhibitor, H3K9me3 accumulates leading to gene repression and decreased HIF-1α mRNA (**E**). In d and e the yellow hexagons represent H3K9me3.

HIF-1α stability is known to be controlled via post-translational modifications involving hydroxylation, acetylation, ubiquitination and phosphorylation^36^. To determine the mechanism behind the reduced levels of HIF-1α protein after KDM4A depletion, we began by analysing the levels of HIF-1α mRNA in cells lacking KDM4A exposed to hypoxia (2 and <0.1% O_2_). Surprisingly, we found that loss of KDM4A led to a significant decrease in HIF-1α mRNA in both normoxia and the milder hypoxic conditions (2% O_2_) (Fig. 4A). A significant decrease in HIF-2αmRNA levels were also observed in cells depleted of KDM4A in hypoxic (2% O_2_) conditions (Supplementary Figure S4A). We did not see a similar effect on HIF-1α mRNA levels when KDM4B or C were depleted (Supplementary Figure S4B,C,D). These data also highlight that HIF-1α mRNA expression is reduced in severely hypoxic conditions (<0.1% O_2_) and that this was not further reduced by loss of KDM4A. A natural antisense of HIF-1α transcript (aHIF-1α), which is complementary to the 3′-untranslated region of the HIF-1α mRNA, has been described to serve as an additional level of HIF-1α transcription regulation^37, 38^. We asked whether KDM4A-depletion could influence the levels of aHIF-1α. We observed that the expression of aHIF-1α increased in response to hypoxia (2% and <0.1% O_2_), but depletion of KDM4A did not have significant effect (Fig. 4B). In addition, depletion of KDM4A did not have a significant effect on the levels of key transcription factors known to play a role in HIF-1α mRNA expression (Supplementary Figure S4E). As shown, depletion of KDM4A leads to an accumulation of both H3K9me3 and H3K36me3, of these, H3K9me3 is associated with heterochromatin and gene repression (Fig. 1B)^39^. This led us to hypothesise that an accumulation of H3K9me3 at the *HIF-1A* gene could explain the loss of HIF-1α expression in response to loss of KDM4A. Using the UCSC genome browser (GRCh37/hg19 genome assembly), we found regions of moderate enrichment of the H3K9me3 chromatin mark along the *HIF-1A* gene (Supplementary Figure S4F). Therefore, we carried out a ChIP assay for H3K9me3 in KDM4A-depleted cells which were incubated in normoxia or hypoxia (2 and <0.1% O_2_) (Fig. 4C). This analysis provided a number of key findings. Firstly, we noticed that H3K9me3 accumulates at the *HIF-1A* gene in response to severe hypoxia (<0.1% O_2_), offering a potential explanation for why HIF-1α mRNA levels decrease in these conditions. Secondly, whilst H3K9me3 did not accumulate at the *HIF-1α* gene in response to 2% O_2_, this was significantly increased by the loss of KDM4A. Finally, loss of KDM4A did not alter the levels of H3K9me3 on the *HIF*-*1A* gene in severe hypoxia (<0.1% O_2_) and this is consistent with the demethylase being inactive in these conditions (Fig. 4D,E). In addition, we observed that depletion of KDM4A led to increased levels of H3K9me3 on the *HIF-1A* gene at 4 other regions (Supplementary Figure S4G).

## Discussion

This study describes a novel role of the KDM4A histone demethylase in regulating HIF-1α expression and activity. Most interestingly, our study describes a novel transcriptional method of HIF-1 regulation. In normal conditions a balance of methyl transferase and demethylase activity regulates repressive marks such as H3K9me3 and therefore gene expression. Here, we showed that when the activity of one such demethylase, KDM4A, is restricted due to depletion of the protein or lack of an essential co-factor, such as oxygen, H3K9me3 accumulates along the *HIF-1A* gene (Fig. 4E). This in turn leads to decreased levels of HIF-1α mRNA and reduced protein stabilisation/activity.

Although inhibition of KDM4A has not undergone clinical testing yet in cancer patients it is widely considered an attractive strategy. Caution is warranted however, as the likely off-target effects of active-site metal-chelating KDM inhibitors include stabilising HIF through direct or indirect (e.g. via altering Fe(II) availability in cells) inhibition of related 2OG oxygenases PHD/FIH. Interestingly, a recent report demonstrated that LSD1 regulates HIF-1α, highlighting further overlap between the histone demethylases activity and HIF^40^. Most importantly, our data suggests that should specific KDM4A inhibitors become available they may well have the previously unforeseen benefit of reducing HIF activity and therefore significantly impact tumour aggressiveness and therapy resistance.

## Methods

### Cell lines, transfections and drug treatments

RKO, MCF7, MDA-MB-231, HCT116 and FaDu^HRE-luc^ were cultured in Dulbecco’s modified Eagle’s medium (D-MEM) supplemented with 10% FBS and 1% penicillin/streptomycin. For siRNA experiments, cells were transfected with 10 nM siRNA using Lipofectamine RNAiMAX in OptiMEM minimal medium (Invitrogen). As a negative control, ON-TARGETplus Non-targetting Control Pool siRNA (Dharmacon, D-001810-10) was used. siKDM4A S1 (Ambion, ID: 148456), siKDM4A Q1-Q4 (Qiagen, 10274116), siKDM4B (Ambion, ID: 148507), siKDM4C (Ambion, ID: 108664), siKDM4D (Dharmacon, siGENOME SMARTpool D-020709-01 to 04). ML324 (Sigma-Aldrich, SML0741), JIB-04 (Sigma-Aldrich, SML0808) and IOX1 (Sigma Aldrich, SML0067).

### Hypoxia treatment

Hypoxic treatments at 2% O_2_ were carried out in a Don Whitley H35 Hypoxystation and at <0.1% O_2_ in a Bactron chamber (Shel Lab). Radiation of cells in hypoxic conditions was carried out as previously described^41^. Values are presented as mean ± SEM of three independent experiments.

### Immunoblotting

Cells were lysed in UTB (9 M urea, 75 mM Tris-HCl pH 7.5, 0.15 M β-mercaptoethanol) and briefly sonicated. Primary antibodies used: HIF-1a (BD Transduction Labs., 610959), KDM4A (Abcam, ab24545), CAIX (BioScience, AB1001), Glut1 (Abcam, ab14683), Actin (Santa Cruz, sc-69879), H3K9me3 (Millipore, 07-422), H3K36me3 (Abcam, ab9050), H3 (Cell Signaling, 36385), NFκB p52 (Millipore, 05-361), Sp1 (Millipore, 07-645), E2F-1 (Cell Signaling, 3742S), HIF-2a (Novus Biologicals, NB100-122). Secondary antibodies were IRDye® 680RD Goat anti-Mouse IgG (H+L), IRDye® 680RD Goat anti-Rabbit IgG (H+L), IRDye® 800CW Donkey anti-Mouse IgG (H+L) and IRDye® 800CW Donkey anti-Rabbit IgG (H+L) from LI-COR Biosciences. Odyssey IR imaging technology (LI-COR Biosciences) was used for imaging.

### qRT-PCR

mRNA was prepared using TRI reagent (Sigma). A NanoDrop was used for quantification. cDNA was synthesised using the Verso cDNA Enzyme kit (Life Technologies). qPCR was carried out with SYBR mix using a Step One Plus Real-time PCR Detection System (Applied Biosystems). All mRNA expression levels are normalised to siCtrl 18S mRNA. Primer sequences are available in the SI. Values are presented as mean ± SEM of three independent experiments.

### HIF reporter assay

Luciferase was measured with Dual-Glo® Luciferase Assay System (Promega). Readings of RKO cells were normalised to Renila Luciferase signal whereas for FaDu^HRE-Luc^ they were normalised to the number of cells in each condition. Values are presented as mean ± SEM of three independent experiments.

### ChIP

As previously described^27^, IgG (Cell Signaling), H3 (Abcam, ab1791), H3K9me3 (Abcam, ab8898) antibodies were used. Primers are listed in SI. Values are presented as mean ± SEM of three independent experiments.

### Measurement of ECAR and OCR

ECAR was measured using a Seahorse Bioscience XF96 Extracellular Flux Analyzer (Agilent Technologies). RKO cells (16000 cells/well) were seeded on polystyrene Seahorse XF Cell Culture Microplates and depleted of KDM4A for 48 h. 24 h before the experiment, cells were incubated in 2% O_2_ for 24 h. The medium was changed to physiological medium (XF Assay Medium with 5 mM Glucose, 5 mM Pyruvate, 4 mM Glutamine, pH 7.4) and measurements immediately acquired. ECAR was calculated by the Seahorse XF96 software and subsequently normalised to cell number, which was quantified by staining the cells with Hoechst and measuring the intensity with POLARstar Omega microplate reader Spectrophotometer (BMG Labtech). Values are presented as mean ± SEM of three independent experiments.

### xCELLigence assay

The xCELLigence Real-Time Cell Analyser (RTCA) DP Instrument equipped with a CIM-plate 16 (Roche) was used as previously^42^. For quantification, the cell index at indicated time points was averaged from three independent experiments.

### Statistical analysis

Statistical significance was calculated using GraphPad Prism software. For qRT-PCR, HIF reporter assay, ChIP and Seahorse results unpaired, two-tailed t-test was used, whereas for xCELLigence and colony formation experiment Two-way ANOVA with Alpha 0.05 was employed.

## Electronic supplementary material

SI
