# Supplementary material for: KDM4A regulates HIF-1 levels through H3K9me3
Source: Sci Rep. 2017 Sep 11;7:11094. doi: 10.1038/s41598-017-11658-3 (PMC5593970; doi:10.1038/s41598-017-11658-3)
Supplement: Supplementary file 1 — SI [file 41598_2017_11658_MOESM1_ESM.pdf]

KDM4A regulates HIF-1 levels through H3K9me3

Grzegorz Dobrynin[1], Tom E. McAllister[3], Katarzyna B. Leszczynska[1], Shaliny Ramachandran[1], Adam J. Krieg[4], Akane Kawamura[2,3] and Ester M. Hammond[1$]

[1]Cancer Research UK and Medical Research Council Oxford Institute for Radiation Oncology, Department of Oncology, The University of Oxford, Oxford, OX3 7DQ, UK. [2]Division of Cardiovascular Medicine, Radcliffe Department of Medicine, Wellcome Trust Centre of Human Genetics, Roosevelt Drive, The University of Oxford, Oxford, OX3 7BN. [3]Department of Chemistry, Chemistry Research Laboratory, The University of Oxford, Mansfield Road, Oxford OX1 3TA, UK. [4]Department of Obstetrics and Gynecology, Oregon Health & Science University, Portland, Oregon, USA.

SUPPLEMENTARY INFORMATION

SI Figure legends Pages 2-7

Table S1 – Primer sequences Page 8

Table S2 – ChIP primers Page 9

Supplemental methods Page 10

Supplementary figures Page 12

SI FIGURE LEGENDS

Figure S1. Biological consequences of siRNA-mediated KDM4A depletion

(**a**) Expression analysis of *KDM4A* and *KDM4B* genes in The Cancer Genome Atlas (TCGA) data sets for colorectal adenocarcinoma. RNA-sequencing data (RNA Seq V2 RSEM) for 382 colorectal adenocarcinoma tumours were downloaded from the TCGA project (accessed through cBioportal: http://www.cbioportal.org/ on the 12th April 2017). To examine KDM4A and KDM4B expression against hypoxia signature [1], raw data for each sequenced gene were rescaled to set the median equal to 1, and hypoxia signature was determined by quantifying the median expression of genes from the hypoxic signature. Log10 conversion of the hypoxia signature was plotted against Log10 conversion of raw data for (**a**) KDM4A or (**b**) KDM4B (also rescaled to set the median equal to 1). Correlations and statistical significance were determined by calculating Spearman's rho (r) rank correlation coefficients and two-tailed *P* values using Hmisc package in RStudio.

(**c**) RKO cells were treated with siRNA targeting KDM4A for 48 h and then incubated in 21%, 2% or <0.1% O_2 for 24 h. Afterwards RNA extraction was performed followed by qRT-PCR for KDM4A.

(**d**) Quantification of the data shown in Figure 1B. The experiment was carried out 3 times and the levels of H3K9me3 and H3K36me3 quantified using Image J. The average values are shown.

(**e**) Schematic representation of the radiation/hypoxia experiments.

(**f**) RKO cells were treated as in Figure 1D, incubated for 24 h and irradiated with the doses indicated, in normoxia (21% O_2)

(**g**), hypoxia (2% O_2)

(**h**) or hypoxia (<0.1% O_2) as previously described [2]. A colony survival assay was then carried out.

(**i**) MDA-MB-231 cells were treated as in Figure 1E and then relative cell motility was determined with xCELLigence Real-Time Cell Analyser (RTCA) DP Instrument equipped with a CIM-plate 16 (Roche) as previously [3]. Efficient knock-down of KDM4A in this cell line is shown in Supplementary Figure S3B.

Values in (c, e, f, g and h) are presented as mean ± SEM of three independent experiments. Statistical significance was calculated using GraphPad Prism software. For qRT-PCR results, unpaired t-test was used, whereas for xCELLigence and colony formation experiment Two-way ANOVA with Alpha 0.05 was employed.

Figure S2. ML324 and IOX-1 stabilise HIF-1α protein levels in normoxic conditions (21% O_2)

(**a**) RKO cells were treated with increasing concentrations (0-10 μM) of ML324 for 6 h. Western blotting was carried out with the indicated antibodies, and the levels of Actin are shown as loading control.

(**b**) RKO cells were treated with ML324 (10 μM) for increasing periods of time and Western blotting was carried out as in (**a**).

(**c**) RKO, OE19, OE21 or OE33 cells were treated with ML324 (10 μM) for 48 h. Western blotting was then carried out as in (**a**).

(**d**) RKO cells were treated with increasing concentrations (0-50 μM) of IOX-1 for 48 h and Western blotting was carried out as in (**a**).

(**e**) RKO cells were treated with ML324 (10 μM) for the times indicated at 21% O_2 and the relative mRNA levels of Glut1 or VEGF were determined by qRT-PCR. mRNA was prepared using TRI reagent (Sigma). qPCR was carried out with SYBR mix using a Step One Plus Real-time PCR Detection System (Applied Biosystems). All mRNA expression levels are normalized to siCtrl 18S mRNA. All primers used for qRT-PCR experiments are presented in Supplementary Table 1.

(**f**) RKO cells were treated with ML324 (10 μM) for the times indicated at 21% or 2% O_2 and the relative mRNA expression levels of VEGF were determined by qRT-PCR. Values are presented as mean ± SEM of three independent experiments. Statistical significance was calculated using unpaired t-test with GraphPad Prism software.

(**g**) IC_{50} determination of inhibitors against PHD2 and FIH using mass spectrometry assay. For PHD2 activity assays, recombinant $PHD2_{181\text{-}426}$ [4] in HEPES (50 mM, pH 7.5) was pre-incubated with inhibitor for 20 min (2 μM enzyme and 2% DMSO) and the reaction initiated by addition of 1 volume of cofactor solution. The final concentrations in the reaction mixture were: Fe(II) (5 μM), 2OG (10 μM), ascorbate (100 μM), HIF-1αCODD(M561A,M568A)$_{556\text{-}574}$ (H-DLDLEALAPYIPADDDFQL-OH, 50 μM), PHD2 (1 μM), DMSO (1% *v/v*)) in 50 mM HEPES (pH 7.5). After 15 minutes at room temperature, the reaction was quenched with 1 volume formic acid (2 % *v*/*v*). The reaction mixture was mixed 1:1 with α-cyano-4-hydrocinnamic acid (CHCA) solution (water: acetonitrile 1:1 + 0.1% TFA) and analyzed by matrix-assisted laser desorption/ionization time-of-flight mass spectrometry (MALDI-TOF MS) using a Waters® Micromass® MALDI micro MX™ mass spectrometer. Data were analyzed using Mass Lynx™ v4.0. For FIH activity assays, recombinant FIH[5] was pre-incubated with inhibitor for 10 minutes (200 nM enzyme and 2% DMSO) and the reaction was initiated by addition of 1 volume of cofactor solution (final

concentrations Fe(II) (10 μM), 2OG (10 μM), ascorbate (100 μM), ankyrin peptide (H-ALEVVKLLLEAGADVNAQDK-OH, 5 μM), FIH (100 nM), DMSO (1% *v/v*)) in buffer (50mM Tris-HCl (pH 7.8 at 22 °C), 50mM NaCl). After 10 minutes at room temperature, reactions were quenched with 1/10 volume formic acid (10% *v/v*). Assay plates in (g) and (h) were transferred onto a RapidFire360 integrated autosampler/solid phase extraction (SPE) coupled to an Agilent 6520 Accurate-Mass Q-TOF mass spectrometer system (Agilent Technologies Inc., Wakefield, MA, USA). Solvent A was water containing 0.1% (v/v) formic acid. Solvent B was acetonitrile/water (8:2, v/v) containing 0.1% (v/v) formic acid. Samples were aspirated under vacuum directly from 384-well assay plates for 500 ms. The sample was then loaded onto a C4 solid phase extraction cartridge to remove non-volatile buffer salts, using solvent A at a flow rate of 1.5 mL/min for 5.5 s. The retained analytes were eluted to the mass spectrometer by washing the cartridge with solvent B at 1.25 mL/min for 4 s. The cartridge was re-equilibrated with solvent A for 500 ms at 1.5 mL/min. Data were integrated and processed using the RapidFire™peak integration software. Assays were carried out in technical triplicates and data were normalized to both a no enzyme negative control and a no inhibitor DMSO positive control. Data were analyzed using GraphPad Prism® 5.0 and fit to four-parameter dose-response curve.

Figure S3. Depletion of KDM4A leads to decreased levels of HIF-1α protein levels in hypoxia (2% O_2)

(**a**) RKO cells were treated with indicated siRNAs for 48 h and incubated in 2% O_2 for 24 h. Western blotting was carried out with the antibodies indicated, and the levels

of Actin are shown as loading control. siKDM4A Q1-Q4 (Qiagen, Cat. No. 10274116).

(**b**) MDA-MB-231 cells were treated with siRNA targeting KDM4A for 48 h and incubated in 21%, 2% or <0.1% O_2 for 24 h. Western blotting was carried out as in part (**a**). HIF-2α (Novus Biologicals, NB100-122).

(**c**) HCT116 cells were treated with siRNA targeting KDM4A for 48 h and incubated in 21%, 2% or <0.1% O_2 for 24 h. Western blotting was carried out as in part (**a**).

RKO cells were treated with siRNA targeting KDM4A for 48 h and then incubated in 21%, 2% or <0.1% O_2 for 24 h. qRT-PCR for Glut1 (**d**), CAIX (**e**) or OAZ1 (**f**). Values in (d, e and f) are presented as mean ± SEM of three independent experiments. Statistical significance was calculated using unpaired t-test with GraphPad Prism software.

Figure S4. Mechanism of KDM4A-dependent effect on HIF-1α

(**a**) RKO cells were treated with siRNA targeting KDM4A for 48 h and then incubated in 21%, 2% or <0.1% O_2 for 24 h. qRT-PCR for HIF-2α is shown.

(**b**) RKO cells were treated with siRNA targeting KDM4A, B (Ambion, siRNA ID: 148507) or C (Ambion, siRNA ID: 108664) for 48 h and then processed as in part (**a**). qRT-PCR for HIF-1α is shown.

RKO cells were treated with siRNA targeting KDM4B for 48 h and then processed as in part (**a**). qRT-PCR results for KDM4B (**c**) or KDM4C (**d**) are shown.

(**e**) RKO cells were treated with siRNA targeting KDM4A for 48 h and incubated in 21%, 2% or <0.1% O_2 for 24 h followed by Western blotting for the indicated

antibodies. NFκB p52 (Millipore, 05-361), Sp1 (Millipore, 07-645), E2F-1 (Cell Signaling, 3742S).

(**f**) Track of H3K9me3 mark along *HIF-1α* from UCSC genome browser (GRCh37/hg19 assembly).

(**g**) RKO cells were treated with siRNA targeting KDM4A for 48 h and then incubated in 2% O_2 for 24 h. H3K9me3 fold enrichment relative to H3 at 5 different locations along the *HIF-1α* gene was assessed by ChIP followed by qPCR for each sample using primers designed to target a region in which H3K9me3 binding was expected (Supplementary Figure 4F). Primers are presented in Supplementary Table 2.

Figure S5. Uncropped blots from the Figure 1.

(**a**) Uncropped blots from the Figure 1A.

(**b**) Uncropped blots from the Figure 1B.

Figure S6. Uncropped blots from the Figure 2.

(**a**) Uncropped blots from the Figure 2A.

(**b**) Uncropped blots from the Figure 2B.

Figure S7. Uncropped blots from the Figure 3.

Uncropped blots from the Figure 3A.

Primer pair HIF-1α 3 forward and reverse were also used in Figure 4C.

Supplementary Table 1. qRT-PCR primers

Name	Forward (5' - 3')	Reverse (5' - 3')
18S	GCCCGAAGCGTTTACTTTGA	TCCATTATTCCTAGCTGCGGTATC
aHIF-1α	TTTGTGTTTGAGCATTTTAATAGGC	CCAGGCCCCTTTGATCAGCTT
Glut1	ATACTCATGACCATCGCGCTAG	AAAGAAGGCCACAAAGCCAAAG
Glut3	ACTTTGACGGACAAGGGAAATG	ACCAGTGACAGCCAACAGG
HIF-1α	TTCACCTGAGCCTAATAGTCC	CAAGTCTAAATCTGTGTCCTG
HIF-2α	TTGATGTGGAAACGGATGAA	GGAACCTGCTCTTGCTGTTC
KDM4A	ATCTAGACTGTCAGTAGCCT	GGTATAGTGCAGGCTCAATA
KDM4B	CGGGTTCTATCTTTGTTTCTCTCACCCG	AAGGAAGCCTCTGGAACACCTG
KDM4C	GGTCAACCCCAACGTGAAGT	CGTTTGACCCACGGAAATG
OAZ1	TTTGGGCTTTGAGATTGTGA	TGAGCGAGTCTACGGTTCCT
SNAI1	ACCACTATGCCGCGCTCTT	GGTCGTAGGGCTGCTGGAA
TWIST1	GGACAAGCTGAGCAAGATTCAGA	TCTGGAGGACCTGGTAGAGGAA
VEGF	CTACCTCCACCATGCCAAGT	CTCGATTGGATGGCAGTAGC
ZEB2	GACCTGGACGTGAAGGAAAA	GGCACTTGCAGAAACACAGA

Supplementary Table 2. ChIP primers

Name	Forward (5' - 3')	Reverse (5' - 3')
HIF-1α 1	TCCCTTCTCCTGGTCCTTCT	AGCACTGTAATCACCTCCGC
HIF-1α 2	TCAGGCCAGACTCCTTCAGA	ACCACCATATGCTGCTTCCT
HIF-1α 3	TTCACAGTCTCCCTTCCCCT	GGGGCCAGCAAAGTTAAAGC
HIF-1α 4	CTCCAGGTTGATCAGGCTGG	GGCTCACAACTGTCATCCCA
HIF-1α 5	TGCATTTGGTTTGTGGGGTG	CCCTGCTCTTCCCACTATGC

SI References

1 Li, B. *et al.* Fructose-1,6-bisphosphatase opposes renal carcinoma progression. *Nature* **513**, 251-255, doi:10.1038/nature13557 (2014).

2 Anbalagan, S. *et al.* Radiosensitization of renal cell carcinoma in vitro through the induction of autophagy. *Radiother Oncol* **103**, 388-393, doi:10.1016/j.radonc.2012.04.001 (2012).

3 Scrace, S., O'Neill, E., Hammond, E. M. & Pires, I. M. Use of the xCELLigence system for real-time analysis of changes in cellular motility and adhesion in physiological conditions. *Methods in molecular biology* **1046**, 295-306, doi:10.1007/978-1-62703-538-5_17 (2013).

4 Chowdhury, R. *et al.* Structural basis for binding of hypoxia-inducible factor to the oxygen-sensing prolyl hydroxylases. *Structure* **17**, 981-989, doi:10.1016/j.str.2009.06.002 (2009).

5 Kelly, L., McDonough, M. A., Coleman, M. L., Ratcliffe, P. J. & Schofield, C. J. Asparagine [small beta]-hydroxylation stabilizes the ankyrin repeat domain fold. *Molecular BioSystems* **5**, 52-58, doi:10.1039/B815271C (2009).

IC_{50} determination of inhibitors against PHD2 and FIH using mass spectrometry assay.

For PHD2 activity assays, recombinant $PHD2_{181\text{-}426}$[4] in HEPES (50 mM, pH 7.5) was pre-incubated with inhibitor for 20 min (2 μM enzyme and 2% DMSO) and the reaction initiated by addition of 1 volume of cofactor solution (final concentrations Fe(II) (5 μM), 2OG (10 μM), ascorbate (100 μM), HIF-1αCODD(M561A,M568A[1])$_{556\text{-}574}$ (H-DLDLEALAPYIPADDDFQL-OH, 50 μM), PHD2 (1 μM), DMSO (1% *v/v*)) in 50 mM HEPES (pH 7.5). After 15 minutes at room temperature, the reaction was quenched with 1 volume formic acid (2 % *v/v*). The reaction mixture was mixed 1:1 with α-cyano-4-hydrocinnamic acid (CHCA) solution (water: acetonitrile 1:1 + 0.1% TFA) and analysed by matrix-assisted laser desorption/ionization time-of-flight mass spectrometry (MALDI-TOF MS) using a Waters® Micromass® MALDI micro MX™ mass spectrometer. Data were analysed using Mass Lynx™ v4.0.

For FIH activity assays, recombinant FIH[5] was pre-incubated with inhibitor for 10 minutes (200 nM enzyme and 2% DMSO) and the reaction was initiated by addition of 1 volume of cofactor solution (final concentrations Fe(II) (10 μM), 2OG (10 μM), ascorbate (100 μM), ankyrin peptide (H-ALEVVKLLLEAGADVNAQDK-OH, 5 μM), FIH (100 nM), DMSO (1% *v/v*)) in buffer (50mM Tris-HCl (pH 7.8 at 22 °C), 50mM NaCl). After 10 minutes at room temperature, reactions were quenched with 1/10 volume formic acid (10% *v/v*).

Assay plates were transferred onto a RapidFire360 integrated autosampler/solid phase extraction (SPE) coupled to an Agilent 6520 Accurate-Mass Q-TOF mass spectrometer system (Agilent Technologies Inc., Wakefield, MA, USA). Solvent A was water containing 0.1% (v/v) formic acid. Solvent B was acetonitrile/water (8:2, v/v) containing 0.1% (v/v) formic acid. Samples were aspirated under vacuum directly from 384-well assay plates for 500 ms. The sample was then loaded onto a C4 solid phase extraction cartridge to remove non-volatile buffer salts, using solvent A at a flow rate of 1.5 mL/min for 5.5 s. The retained analytes were eluted to the mass spectrometer by washing the cartridge with solvent B at 1.25 mL/min for 4 s. The cartridge was re-equilibrated with solvent A for 500 ms at 1.5 mL/min. Data were integrated and processed using the RapidFire™peak integration software.

[1] The native methionine residues were substituted with alanine to eliminate the possibility of methionine oxidation, which would give rise to the same change in mass as hydroxylation.

Assays were carried out in technical triplicates and data were normalized to both a no enzyme negative control and a no inhibitor DMSO positive control. Data were analysed using GraphPad Prism® 5.0. and fit to four-parameter dose-response curve

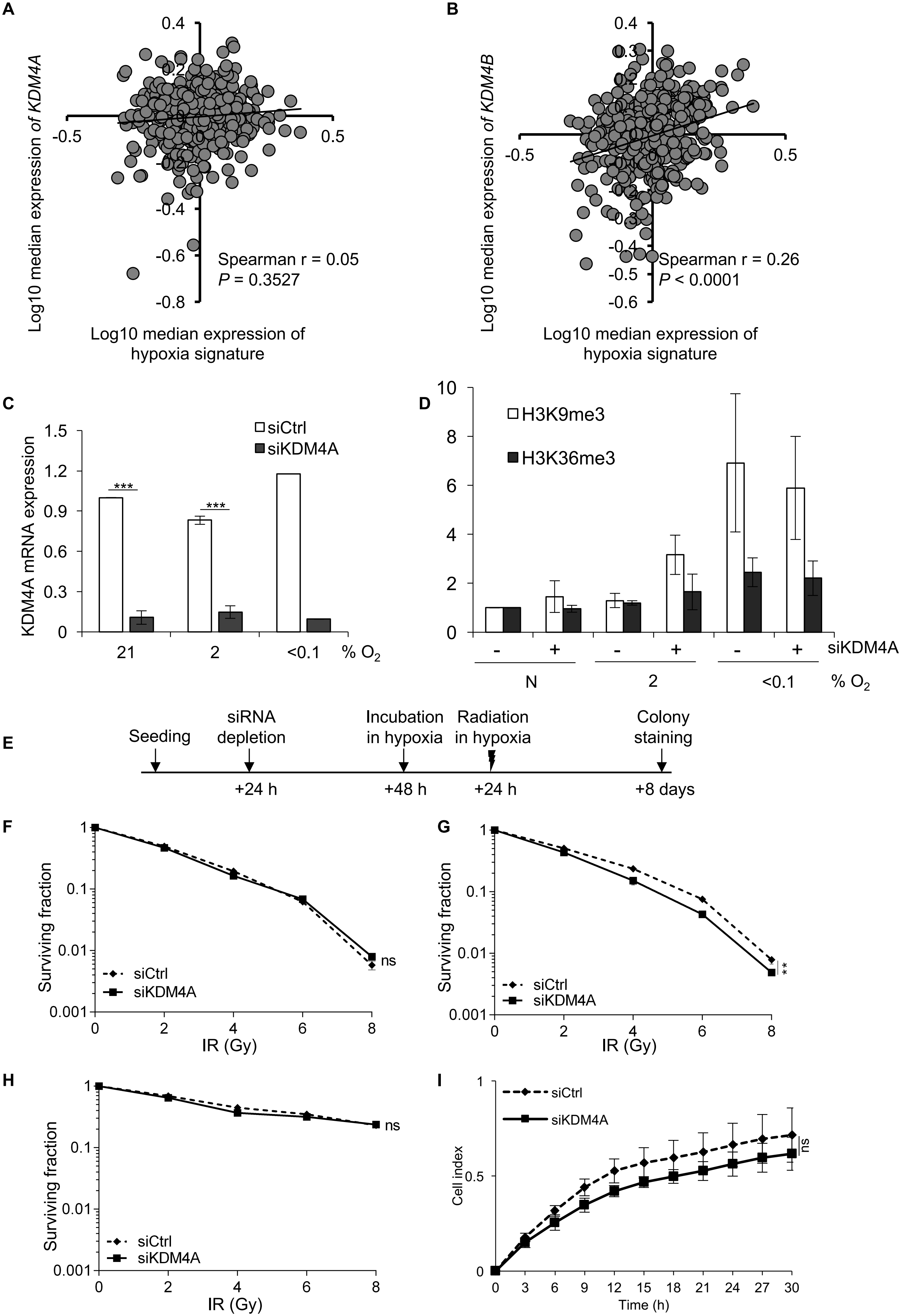

A
Log10 median expression of KDM4A
Log10 median expression of hypoxia signature
Spearman r = 0.05
P = 0.3527
B
Log10 median expression of KDM4B
Log10 median expression of hypoxia signature
Spearman r = 0.26
P < 0.0001
C
KDM4A mRNA expression
siCtrl
siKDM4A
21
2
<0.1
% O2
D
H3K9me3
H3K36me3
siKDM4A
N
2
<0.1
% O2
E
Seeding
siRNA depletion
Incubation in hypoxia
Radiation in hypoxia
Colony staining
+24 h
+48 h
+24 h
+8 days
F
Surviving fraction
IR (Gy)
siCtrl
siKDM4A
ns
G
Surviving fraction
IR (Gy)
siCtrl
siKDM4A
**
H
Surviving fraction
IR (Gy)
siCtrl
siKDM4A
ns
I
Cell index
Time (h)
siCtrl
siKDM4A
ns

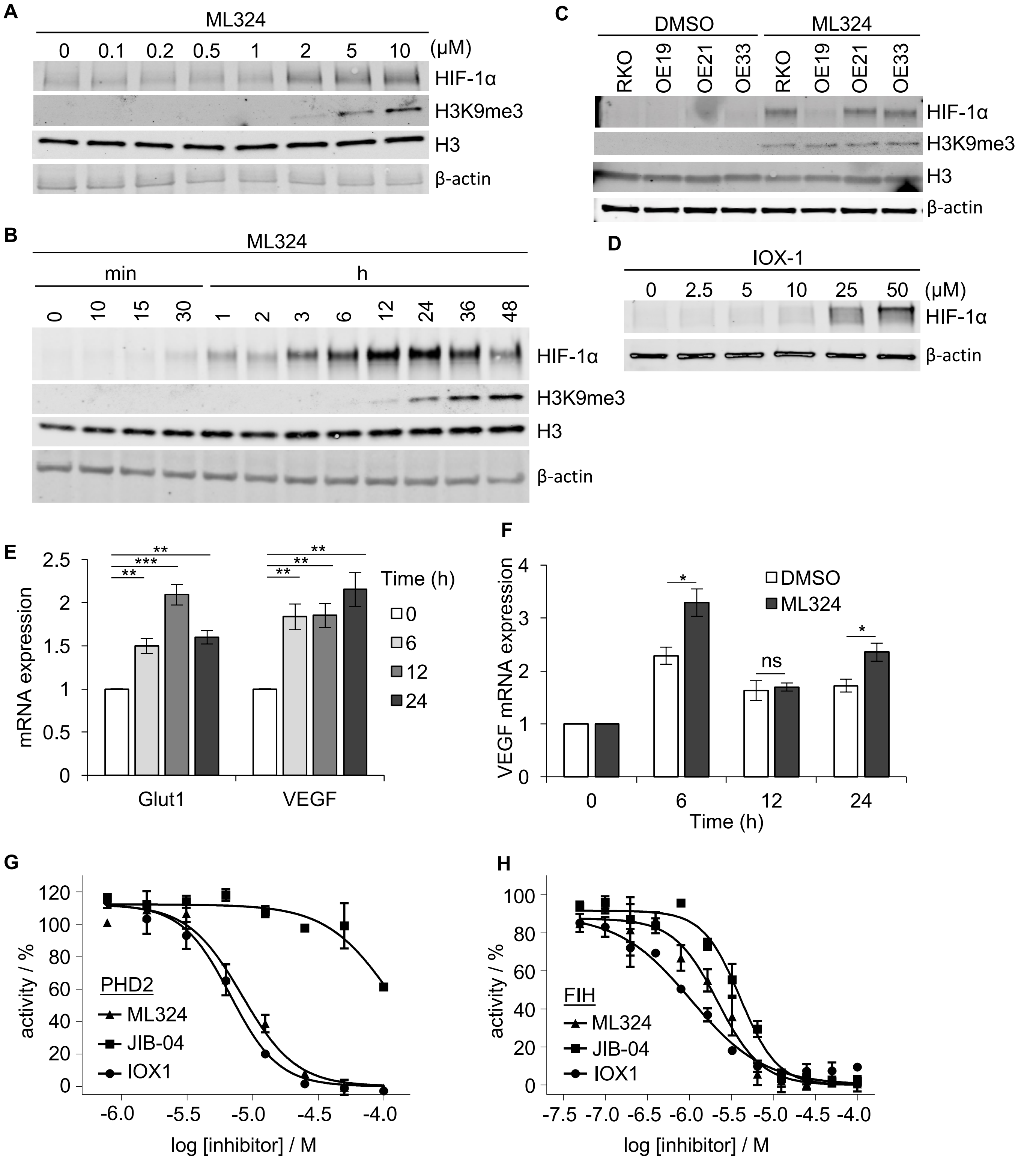
A
ML324
0
0.1
0.2
0.5
1
2
5
10
(μM)
HIF-1α
H3K9me3
H3
β-actin
B
ML324
min
h
0
10
15
30
1
2
3
6
12
24
36
48
HIF-1α
H3K9me3
H3
β-actin
C
DMSO
ML324
RKO
OE19
OE21
OE33
RKO
OE19
OE21
OE33
HIF-1α
H3K9me3
H3
β-actin
D
IOX-1
0
2.5
5
10
25
50
(μM)
HIF-1α
β-actin
E
mRNA expression
Time (h)
0
6
12
24
Glut1
VEGF
F
VEGF mRNA expression
DMSO
ML324
ns
Time (h)
G
activity / %
PHD2
ML324
JIB-04
IOX1
log [inhibitor] / M
H
activity / %
FIH
ML324
JIB-04
IOX1
log [inhibitor] / M

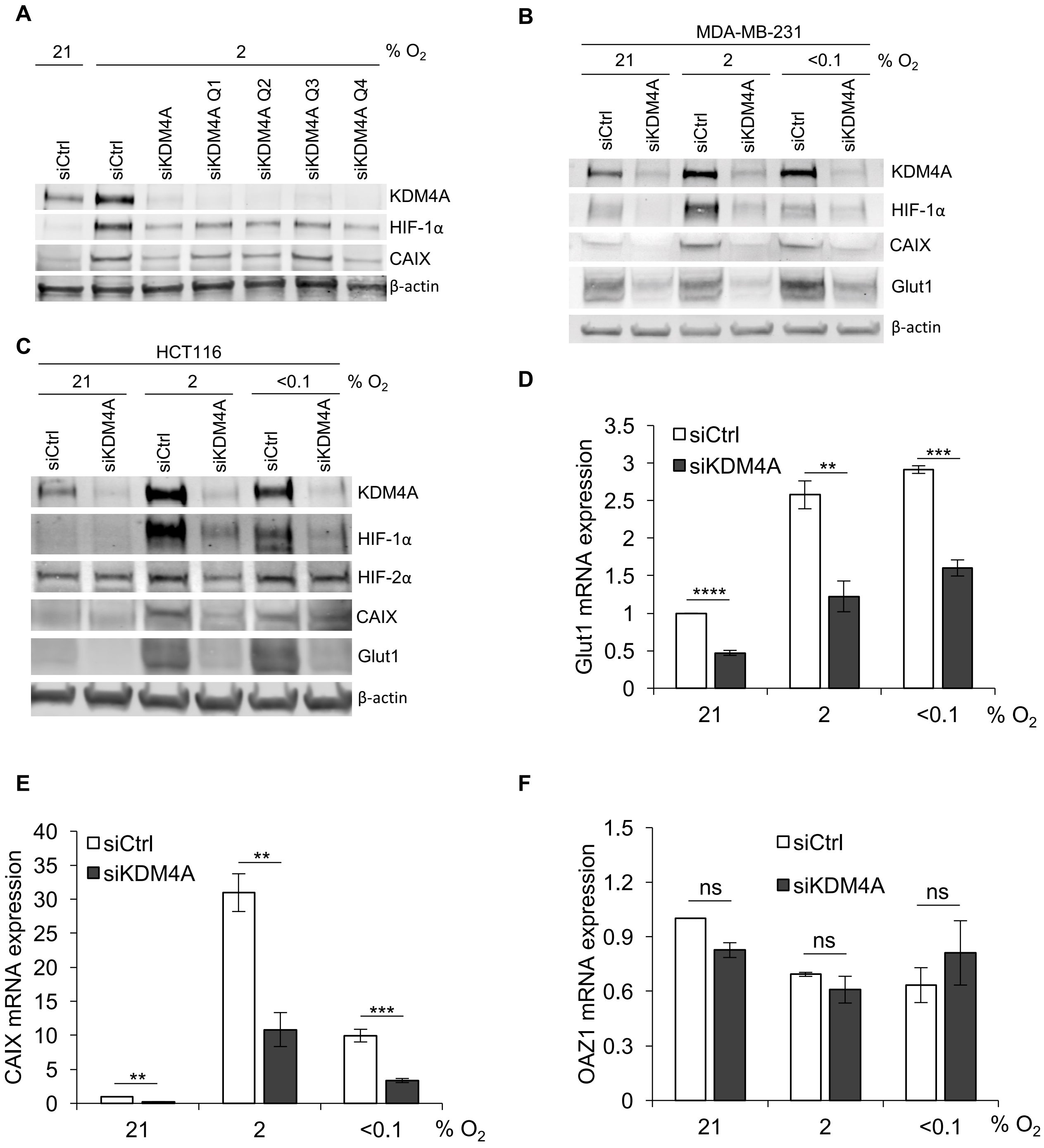
A
21
2
% O2
siCtrl
siCtrl
siKDM4A
siKDM4A Q1
siKDM4A Q2
siKDM4A Q3
siKDM4A Q4
KDM4A
HIF-1α
CAIX
β-actin
B
MDA-MB-231
21
2
<0.1
% O2
siCtrl
siKDM4A
siCtrl
siKDM4A
siCtrl
siKDM4A
KDM4A
HIF-1α
CAIX
Glut1
β-actin
C
HCT116
21
2
<0.1
% O2
siCtrl
siKDM4A
siCtrl
siKDM4A
siCtrl
siKDM4A
KDM4A
HIF-1α
HIF-2α
CAIX
Glut1
β-actin
D
siCtrl
siKDM4A
Glut1 mRNA expression
3.5
3
2.5
2
1.5
1
0.5
0
****
**
***
21
2
<0.1
% O2
E
siCtrl
siKDM4A
CAIX mRNA expression
40
35
30
25
20
15
10
5
0
**
**
***
21
2
<0.1
% O2
F
siCtrl
siKDM4A
OAZ1 mRNA expression
1.5
1.2
0.9
0.6
0.3
0
ns
ns
ns
21
2
<0.1
% O2

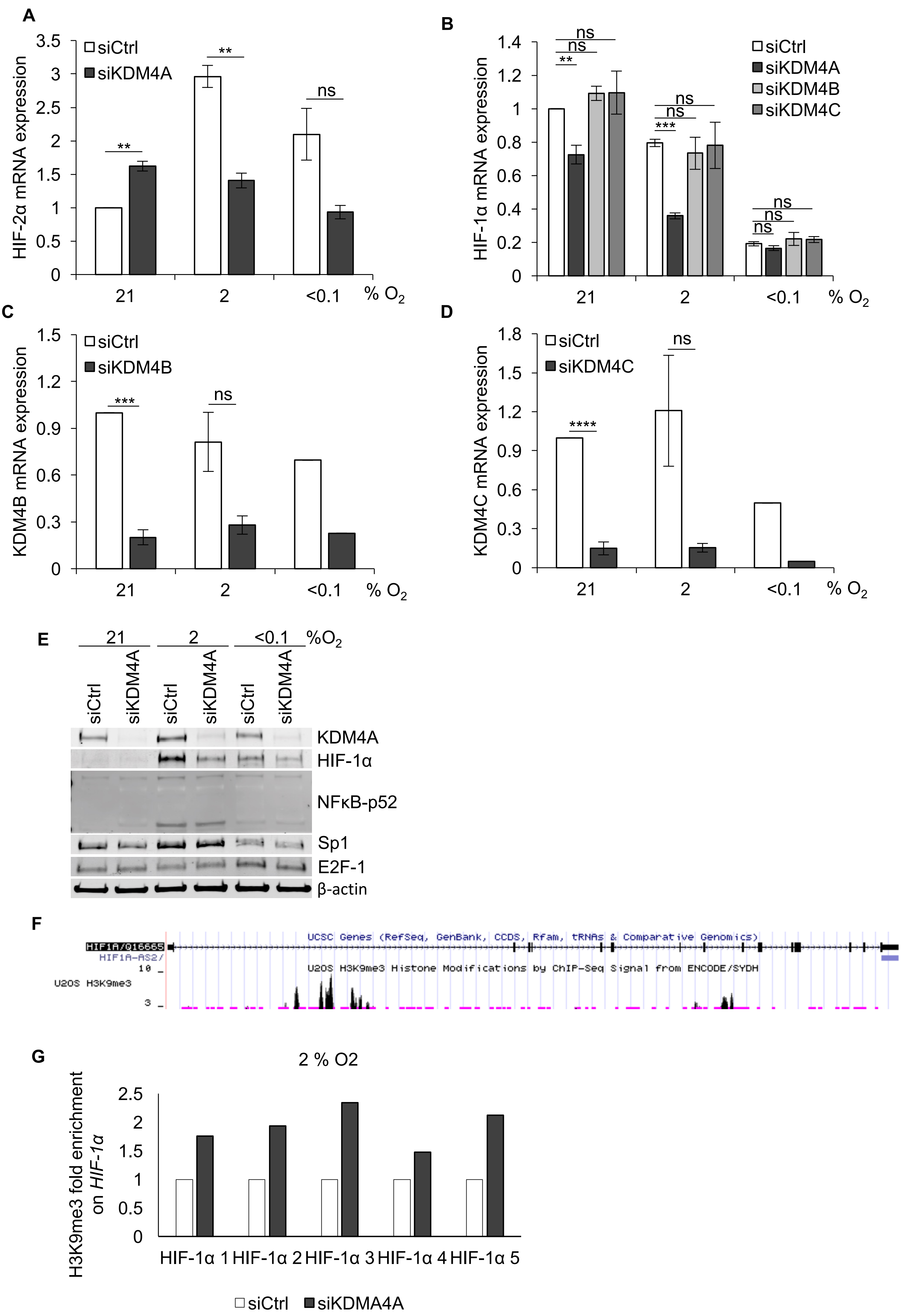
A
HIF-2α mRNA expression
siCtrl
siKDM4A
**
**
ns
21
2
<0.1
% O2
B
HIF-1α mRNA expression
siCtrl
siKDM4A
siKDM4B
siKDM4C
ns
ns
**
***
C
KDM4B mRNA expression
siKDM4B
***
D
KDM4C mRNA expression
siKDM4C
****
E
21
2
<0.1
%O2
siCtrl
siKDM4A
KDM4A
HIF-1α
NFκB-p52
Sp1
E2F-1
β-actin
F
HIF1A/Q16665
HIF1A-AS2/
UCSC Genes (RefSeq, GenBank, CCDS, Rfam, tRNAs & Comparative Genomics)
U2OS H3K9me3 Histone Modifications by ChIP-Seq Signal from ENCODE/SYDH
U2OS H3K9me3
G
2 % O2
H3K9me3 fold enrichment on HIF-1α
HIF-1α 1
HIF-1α 2
HIF-1α 3
HIF-1α 4
HIF-1α 5
siCtrl
siKDMA4A

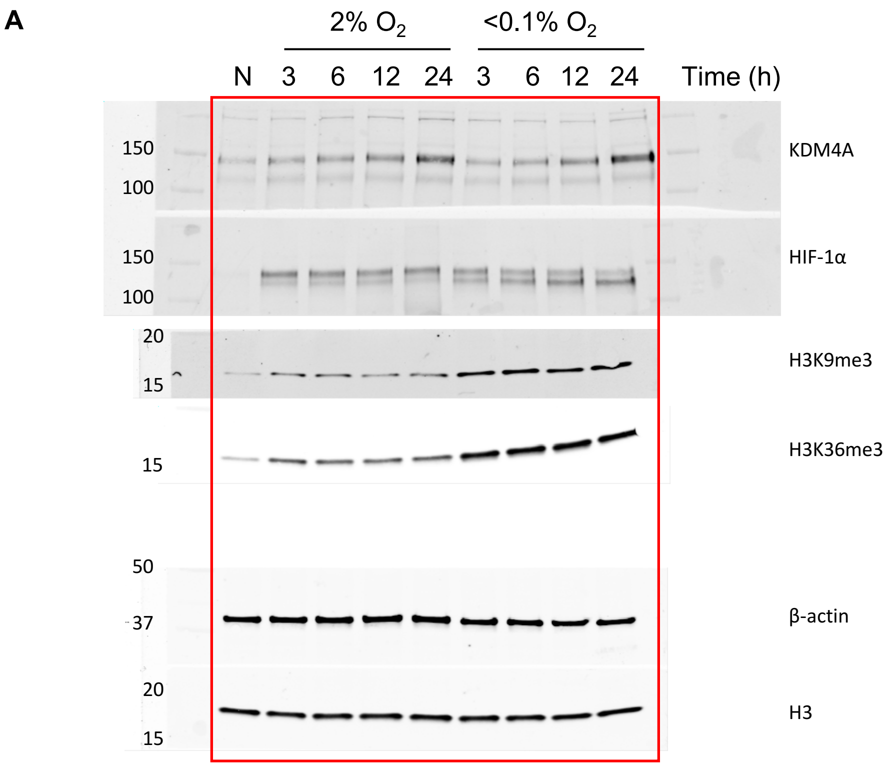
A
2% O2
<0.1% O2
N 3 6 12 24 3 6 12 24 Time (h)
150
100
KDM4A
150
100
HIF-1α
20
15
H3K9me3
15
H3K36me3
50
37
β-actin
20
15
H3

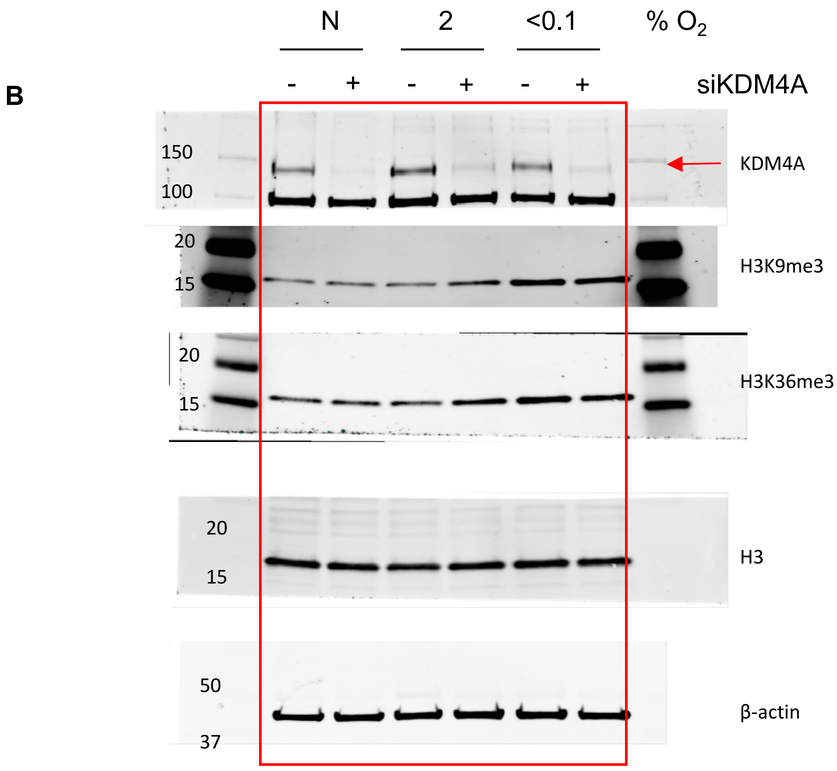
B
N
2
<0.1
% O2
- + - + - + siKDM4A
150
100
KDM4A
20
15
H3K9me3
20
15
H3K36me3
20
15
H3
50
37
β-actin

A

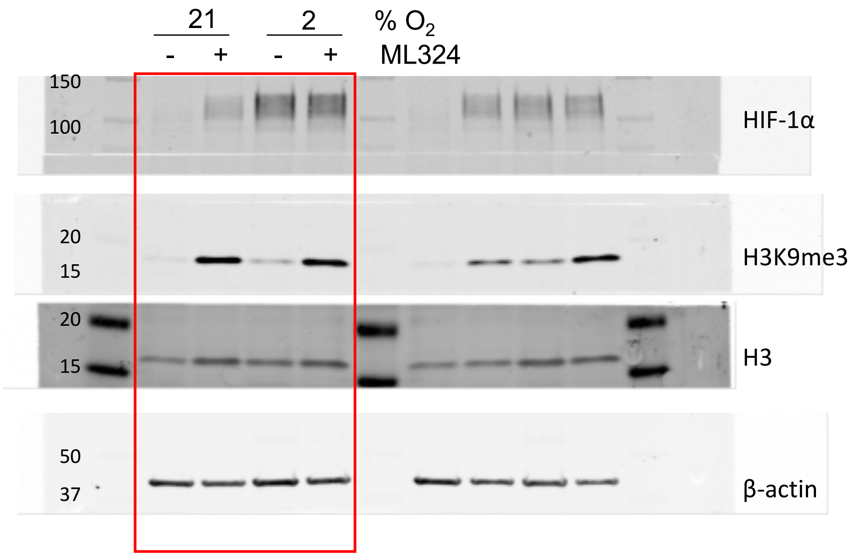

21
2
% O2
- + - +
ML324
150
100
HIF-1α
20
15
H3K9me3
20
15
H3
50
37
β-actin

B

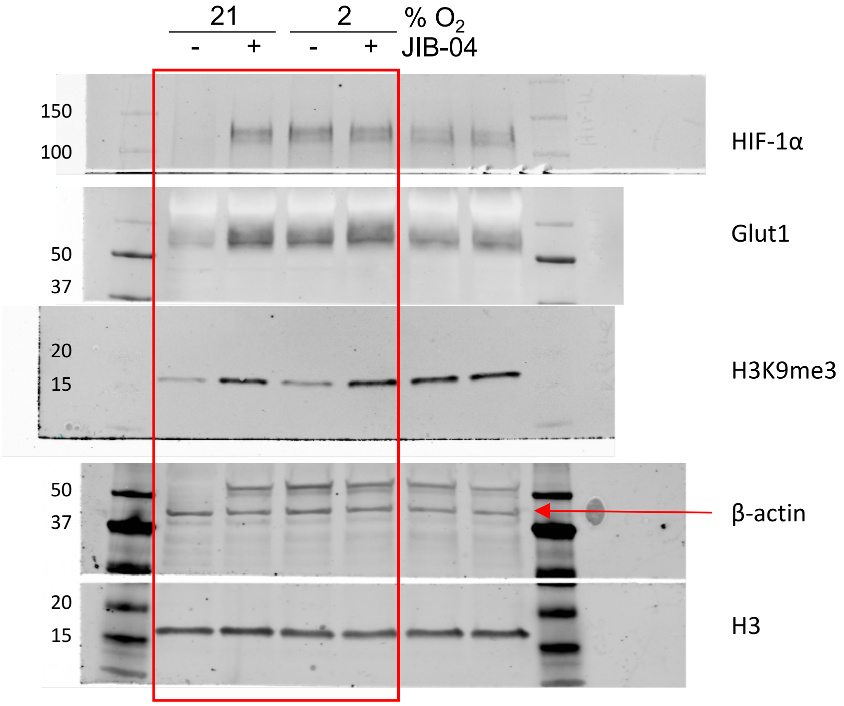

21
2
% O2
- + - +
JIB-04
150
100
HIF-1α
Glut1
50
37
20
15
H3K9me3
50
37
β-actin
20
15
H3

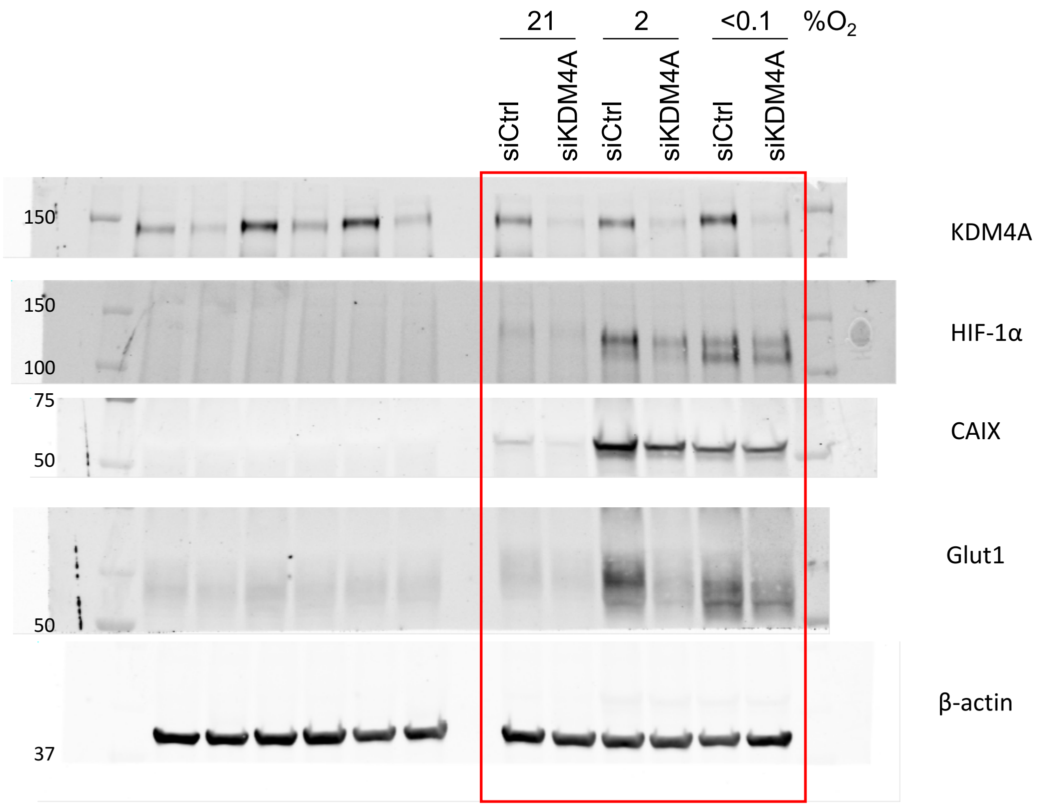

21
2
<0.1
%O2
siCtrl
siKDM4A
siCtrl
siKDM4A
siCtrl
siKDM4A
150
KDM4A
150
100
HIF-1α
75
50
CAIX
50
Glut1
37
β-actin
